# Prevalence and risk factors for nephrolithiasis in adults with cystic fibrosis: A retrospective cohort study

**DOI:** 10.1371/journal.pone.0340293

**Published:** 2026-01-06

**Authors:** Da Yeon Ryoo, Nolan Ladd, Stacey Culp, Spencer Harris, Gretchen Murray, Mariel McGuinness, Stephen E. Kirkby, Ganesh Shidham, Anne Rice, Kristen M. Roberts, Georgios I. Papachristou, Phil A. Hart, Mitchell L. Ramsey

**Affiliations:** 1 Division of Gastroenterology, Hepatology, and Nutrition, College of Medicine, The Ohio State University Wexner Medical Center, Columbus, Ohio, United States of America; 2 Department of Biomedical Informatics, Center for Biostatistics, College of Medicine, The Ohio State University Wexner Medical Center, Columbus, Ohio, United States of America; 3 The School of Health and Rehabilitation Sciences, The Ohio State University Wexner Medical Center, Columbus, Ohio, United States of America; 4 Division of General Internal Medicine, The Ohio State University Wexner Medical Center, Columbus, Ohio, United States of America; 5 Division of Pediatric Pulmonology, Nationwide Children’s Hospital, Columbus, Ohio, United States of America; 6 Division of Nephrology, The Ohio State University Wexner Medical Center, Columbus, Ohio, United States of America; University of Health Sciences, Beyhekim Training and Research Hospital, TÜRKIYE

## Abstract

**Background:**

Calcium oxalate nephrolithiasis is more common in persons living with cystic fibrosis (PwCF) than in the general population. A primary risk factor is exocrine pancreatic insufficiency (EPI) causing enteral hyperoxaluria. However, the relationship between nephrolithiasis and degree of EPI control has not been assessed.

**Methods:**

A retrospective cohort study was conducted including all PwCF seen at our institution from 2018 to 2023 (n = 332). Collected data included socio-demographics, Charlson comorbidity index, EPI control status adjudicated by treating clinicians (classified as controlled, uncontrolled, or unknown), and nephrolithiasis events, which were classified as symptomatic or asymptomatic when nephrolithiasis were discovered incidentally on imaging. Incidence and lifetime prevalence rates were calculated for the entire sample and according to EPI control. A multivariable logistic regression model for the outcome of nephrolithiasis was performed using likelihood-ratio-based backwards stepwise selection.

**Results:**

The cumulative lifetime prevalence of any nephrolithiasis event in our study population was 32%. The lifetime prevalence was higher in EPI (32%) than no EPI (24%), but there was no significant difference in prevalence according to EPI control status. The multivariable model identified that small bowel resection (odds ratio [OR] 3.0, 95% confidence interval (CI) 1.0–9.0)), alcohol use (OR 1.7, 95% CI 1.0–2.9), and decreased BMI (OR 0.9, 95% CI 0.9–1.0) were independently associated with nephrolithiasis. Among 105 individuals with nephrolithiasis, 51% (n = 54) had recurrent events. An invasive procedure was required by 10% (n = 3) with a single nephrolithiasis event and was required by 61% (n = 33) with recurrent nephrolithiasis.

**Conclusions:**

Nephrolithiasis events are common in PwCF. We observed an increased prevalence among PwCF with EPI, but did not observe a difference based on the degree of EPI control although this may be limited by reliance on clinician assessment of EPI control rather than a more objective measure. Prospective investigation with detailed assessment of risk factors including dietary habits and EPI control is warranted.

## Introduction

Nephrolithiasis is estimated to occur in approximately 5% of persons living with cystic fibrosis (PwCF) [1]. However, several groups have reported much higher frequencies, including an Italian cohort reporting a lifetime prevalence of calcium oxalate nephrolithiasis >20% [2]. Among PwCF with nephrolithiasis, nearly 40% required surgical intervention for removal, and nearly 50% developed recurrence [1]. These data show that despite the low overall prevalence of nephrolithiasis among PwCF, the burden on patients and the healthcare system is high.

Calcium oxalate nephrolithiasis is exclusively reported among PwCF with EPI, and is most common among individuals with EPI and essential fatty acid deficiency, which is a marker of poorly controlled EPI [3]. Calcium oxalate nephrolithiasis among PwCF results from enteric hyperoxaluria, where malabsorbed fat in the gastrointestinal lumen binds to calcium and permits increased reabsorption of oxalate [2,4,5]. Accordingly, reducing the amount of malabsorbed fat in the gastrointestinal tract is a first step in management of enteric hyperoxaluria [6,7]. Thus, improving EPI control is expected to reduce the risk of nephrolithiasis, however the risk of nephrolithiasis has not been assessed according to the degree of EPI control. In this study, we address this research gap by assessing the incidence and lifetime prevalence of nephrolithiasis in CF according to the degree of EPI control.

We hypothesized that PwCF with different degrees of EPI control would have variable risk of nephrolithiasis, with increased nephrolithiasis burden seen among subjects with the worst EPI control. While EPI control status is hypothesized to be the primary risk factor for nephrolithiasis in CF, we sought to assess whether age, medication use, or other comorbidities might contribute to the risk of nephrolithiasis independent of EPI status. Lastly, we sought to describe the various treatments offered to PwCF who experience kidney stones.

## Methods

This study was reviewed and approved by The Ohio State University Wexner Medical Center Institutional Review Board (IRB) and was conducted according to the principles in the Declaration of Helsinki. A waiver of written informed consent was granted by the IRB due to the minimal risk of the study design. A retrospective cohort study was conducted at our institution, including all adults with a diagnosis of CF evaluated consecutively between July 1, 2018 and June 30, 2023. The charts of these individuals were reviewed as far back as detailed records were available (generally around 2012 or 2013), with this earliest date considered to be the start of observation. Review of individual participants’ medical records for the purposes of data collection occurred from March 2024 to September 2024. Subjects with CF with less than 2 clinical encounters that each included a full review of symptoms and an assessment of EPI control were excluded (n = 11). A diagnosis of CF (inclusion criterion) was confirmed by review of genetics, sweat chloride results, and multi-organ involvement.

### Definitions

Collected data included socio-demographics, Charlson comorbidity index (without inclusion of the age variable to avoid collinearity in multivariable models), and history of lung transplant. Additional known nephrolithiasis risk factors were abstracted, including smoking status and history of small bowel resection, as well as comorbidities including inflammatory bowel disease, end stage renal disease (ESRD), HTN, and CF-related diabetes. Time varying variables such as age, EPI control status, alcohol use (dichotomized as any prior alcohol use or no prior alcohol use), and renal function were adjudicated near the time of first lifetime nephrolithiasis for those with nephrolithiasis and at the most recent clinical encounter for those without nephrolithiasis.

EPI control status was adjudicated by treating clinicians considering steatorrhea, weight change, and fat-soluble vitamin levels and was classified as controlled, uncontrolled, or unknown. Controlled EPI status used terminology such as “no complaints” and “denies steatorrhea/diarrhea.” Uncontrolled EPI status was determined by terminology such as “complains of steatorrhea/diarrhea” and “reports weight loss” that was otherwise unexplained. Subgroup analyses were performed by including subjects with pancreatic sufficiency in the “controlled EPI” group. The clinician managing EPI was recorded as CF clinic, primary care/internist, gastroenterology, or unknown. Objective markers of EPI control status were sought, but only 13 subjects overall had a recorded fecal fat value. Lastly, units of lipase per kilogram of body weight per day was recorded, calculated based on most recent clinic note and assuming 3 meals and 2 snacks per day.

Nephrolithiasis events were defined as any occurrence of nephrolithiasis on imaging (CT, ultrasound, or X-ray) and was determined based on review of abdominal imaging reports and clinical documentation. Asymptomatic nephrolithiasis was defined as nephrolithiasis on imaging without abdominal or flank pain or urinary tract symptoms. Symptomatic nephrolithiasis was defined by presence of nephrolithiasis on imaging and pain (abdominal or flank) and/or lower urinary tract symptoms (dysuria, hematuria, or urgency). Outcomes included the age at first nephrolithiasis event, type of nephrolithiasis, presence of symptoms at time of nephrolithiasis, and BMI at the time of nephrolithiasis. The total number of events throughout the observation period was recorded. Lastly, treatments for nephrolithiasis were recorded, including medications (citrate supplements, alpha blockers, or diuretics), invasive therapies like ureteroscopy with laser lithotripsy or extracorporeal shock wave lithotripsy (ESWL), and referrals to urology or nephrology. A recurrent nephrolithiasis event was defined by a separate nephrolithiasis episode, unique to the index nephrolithiasis event based on imaging (such as a stone on the contralateral side or proximal to a previously identified stone) with or without symptoms.

### Statistical analysis

Incidence and lifetime prevalence rates were calculated for the entire sample and by EPI control status group. Descriptive statistics were reported for demographic and clinical characteristics. Chi-square tests of independence and Fisher’s exact tests were used, as appropriate based on type of variable (categorical vs continuous) and normality, to compare the distribution of categorical demographic and clinical characteristics between patients with vs. without nephrolithiasis and also between patients with recurrent vs. single nephrolithiasis. Independent samples t-tests (Mann-Whitney U tests) were used to evaluate the difference in means (medians) between groups for continuous variables. Multivariable logistic regression was used to identify factors associated with nephrolithiasis. The initial model included demographics and comorbidities associated with prior nephrolithiasis in the univariable analysis (BMI, Charlson Comorbidity Index (without age), diabetes, alcohol use ever, small bowel resection, lung transplant, and eGFR category) and also age and hypertension. A likelihood-ratio-based backwards stepwise procedure using alpha = 0.10 for variable elimination was used for model selection, resulting in the final model. Data analysis was performed using R version 4.3.1 (Vienna, Austria) and IBM SPSS Statistics version 28 (Armonk, NY).

## Results

### Incidence and prevalence

A total of 332 adults with CF met eligibility criteria for the study cohort. Among these were 107 (32%) with at least one nephrolithiasis event during their lifetime (Table 1). The overall incidence of any nephrolithiasis event was 34 cases per 1,000 person-years during the observation period. PwCF with EPI experienced more nephrolithiasis than those without EPI, but there was no difference between controlled and uncontrolled EPI.The highest incidence and prevalence were among those with EPI and unknown EPI control status.

**Table 1:** Incidence and lifetime prevalence of nephrolithiasis among adults living with CF stratified by exocrine pancreatic insufficiency (EPI) status and EPI control status.

	N	Person-years of follow up	Number of new Cases	Incidence per 1,000 person-years	Number ofcases	Lifetime prevalence
**Overall**	**332**	**3033.86**	**102**	**33.6**	**107**	**32.2%**
No EPI	38	263.94	7	26.5	9	23.7%
EPI (Controlled + Uncontrolled)	257	2418.96	78	32.3	81	31.5%
**EPI Control Status Unknown	37	350.97	17	48.4	17	45.9%

Exocrine pancreatic insufficiency (EPI).

*New cases are defined as participants with a new nephrolithiasis event during the observation period. Prevalent cases are defined as participants with a nephrolithiasis event before or during the observation period start date.

**EPI control is distinct from EPI presence/absence, and is intended to capture day-to-day symptoms related to the adequate use of PERT to control EPI symptoms. All of these individuals with unknown degree of EPI control have EPI, but the day-to-day symptoms could not be assessed retrospectively based on available documentation.

### Features associated with nephrolithiasis among PwCF

Subjects with nephrolithiasis had a lower BMI, but otherwise had similar age and sex distribution (Table 2). Additionally, those with nephrolithiasis had a greater number of comorbidities, including CF-related diabetes and hyperparathyroidism, and were more likely to have had a lung transplant or small bowel resection. There was no significant difference in the proportion of subjects with EPI, degree of EPI control, PERT dose, or PPI use between groups. The specialty of the clinician prescribing PERT varied according to group, with the CF team managing more of the individuals with no prior kidney stones and gastroenterology or other providers prescribing PERT for individuals with prior kidney stones. The multivariable regression model identified low BMI, alcohol use, and a history of small bowel resection as features independently associated with nephrolithiasis (Table 3). PwCF with a prior lung transplant were more likely to have asymptomatic nephrolithiasis, receive medication to prevent recurrence, and be referred to nephrology compared to those without lung transplant (S1 Table).

**Table 2:** Comparison of demographic and clinical characteristics between PwCF with and without prior nephrolithiasis.

Variable	Prior Nephrolithiasis N=107 (32.2%)	No Nephrolithiasis N=225 (67.8%)	p-value
**Demographics**			
Age, years, Mean (SD)	38.8 (13.3)	36.8 (13.0)	0.194
Sex, Male, n (%)	58 (52.4)	112 (49.8)	0.451
Race, White, n (%)	99 (97.1)	205 (97.2)	1.000
Ethnicity, Not Hispanic or Latino, n (%)	102 (100)	203 (98.5)	0.553
BMI			
Mean (SD)	23.0 (4.3)	24.7 (4.9)	0.004
Duration of observation, years			
Median (IQR)	10.6 (7.3–12.1)	9.2 (5.2–11.8)	0.090
Alcohol use	77 (72.0)	130 (57.8)	0.013
Smoking	15 (14.0)	28 (12.4)	0.690
**Comorbidities**			
Charlson comorbidity index (minus age)			
Median (IQR)	1 (0–2)	0 (0–1)	< 0.001
Diabetes	61 (57.0)	82 (36.4)	< 0.001
Hypertension	21 (19.8)	32 (14.2)	0.196
End stage renal disease (ESRD)	5 (4.7)	6 (2.7)	0.342
Hyperparathyroidism	5 (4.7)	0 (0)	0.003
Inflammatory bowel disease (IBD)	1 (0.9)	0 (0)	0.322
History of small bowel resection	9 (8.4)	7 (3.1)	0.035
History of lung transplant	20 (18.7)	18 (8.0)	0.004
Estimated glomerular filtration rate (eGFR)			0.016**
< 30	7 (6.5)	3 (1.4)	
30 – 59.9	5 (4.7)	9 (4.1)	
60+	95 (88.8)	208 (94.5)	
**EPI-related variables**			
EPI present	98 (91.6)	196 (87.1)	0.231
Age at EPI diagnosis	26.3 (10.9)	23.1 (12.3)	0.038
EPI Status, n (%)			
Controlled	71 (66.4)	152 (67.6)	0.204
Uncontrolled	10 (9.3)	24 (10.7)	
Unknown control	17 (15.9)	20 (8.9)	
Normal Exocrine	9 (8.4)	29 (12.9)	
Function/No EPI			
EPI manager			0.015
Gastroenterology	11 (11.2)	20 (10.2)	
Internist	4 (4.1)	4 (2.0)	
Cystic Fibrosis Clinic	58 (59.2)	150 (76.5)	
Other	12 (12.2)	12 (6.1)	
Unknown	13 (13.3)	10 (5.1)	
Pancreas enzyme replacement dose* (units of lipase per kilogram per day)	6858 (3028)	6407 (2224)	0.149
Proton pump inhibitor use	77 (72.0)	146 (64.9)	0.200

*Among subjects with EPI (n = 294), this was calculated according to PERT prescription assuming 3 meals and 2 snacks per day divided by weight in kilograms.

**For eGFR < 30 vs > 30.

**Table 3:** Multivariable logistic regression results for the outcome of prior nephrolithiasis.

Variable	Odds Ratio	OR 95% CI	p-value
BMI (per 1 unit (kg/m^2^)increase)	0.930	(0.880, 0.983)	0.010
CCI (excluding age) (per 1 unit increase)	1.223	(0.986, 1.516)	0.067
Diabetes	1.635	(0.913, 2.929)	0.098
Alcohol Use	1.711	(1.014, 2.886)	0.048
History of small Bowel Resection	3.031	(1.021, 8.997)	0.045

### Recurrent nephrolithiasis among PwCF

Among 105 individuals with nephrolithiasis and sufficient follow up time to evaluate interventions 51% (n = 54) had recurrent events during observation. Among those with recurrences, the median number of lifetime nephrolithiasis events was 3, but 14 individuals were noted to have 10 or more lifetime nephrolithiasis events. Among the 39 stones which were retrieved for analysis, 35 (89.7%) were calcium oxalate and 4 (10.3%) were other types.

In a subgroup analysis, age and BMI at first event were similar between subjects with single or recurrent nephrolithiasis (Table 4). Subjects with recurrent nephrolithiasis were nearly twice as likely to receive an intervention (82% vs 45%, p < 0.001), with higher rates of PERT adjustment, prescription medications to prevent recurrent nephrolithiasis, increased fluid intake goals, and referrals to nephrology or urology. Subjects with recurrent nephrolithiasis were more likely to receive an invasive procedure (61% vs 10%, p < 0.001).

**Table 4:** Comparison of clinical factors in subjects with a single vs. recurrent episodes of nephrolithiasis.

Variable	Single event N = 51	Recurrent events N = 54	p-value
Age at first event, years, Mean (SD)	31.4 (8.5)	30.3 (13.4)	0.632
BMI at time of first event, years, Mean (SD)	22.6 (4.1)	23.9 (6.8)	0.296
Symptomatic	22 (45.8)	45 (84.9)	< 0.001
Type of Nephrolithiasis*			
Calcium oxalate	8 (100)	27 (87.1)	0.563
Other	0 (0)	4 (12.9)	
Any Intervention	23 (45.1)	44 (81.5)	< 0.001
Type of intervention			
Pancreas enzyme replacement therapy dose adjustment	0 (0)	5 (9.4)	0.057
Addition of medication to prevent recurrence	19 (37.3)	31 (57.4)	0.039
Citrate supplement	16 (31.4)	26 (48.1)	0.079
Increased fluid goal and/or diet change	8 (15.7)	23 (43.4)	0.002
Invasive procedure	3 (9.8)	33 (61.1)	< 0.001
Referral to nephrology	2 (3.9)	21 (38.9)	< 0.001
Referral to urology	13 (25.5)	39 (72.2)	< 0.001
24hr urine ever	4 (8.0)	12 (23.1)	0.036

*Includes n=39 with retrieved stones.

## Discussion

As PwCF are living longer with reduced pulmonary symptoms, non-pulmonary manifestations of CF are emerging as important contributors to the patient experience in CF. In this report, we assessed the incidence and prevalence of nephrolithiasis in a large cohort of adults with CF. In contrast to prior studies showing a prevalence between 5–10%, nephrolithiasis events were much more common in our population, with nearly 1/3 of adults with CF experiencing at least one nephrolithiasis event. Additionally, approximately half of individuals will have recurrent episodes, often leading to invasive procedures. We identified alcohol use as an independent modifiable risk factor for nephrolithiasis in PwCF as well as another risk factor (prior small bowel resection) that contributes to the pathophysiology of enteral hyperoxaluria. We hypothesized that EPI control status would be the primary determinant of nephrolithiasis risk, but did not observe a difference in EPI control status between those with and without nephrolithiasis. Additional study is needed to further identify modifiable risk factors for nephrolithiasis among PwCF in order to minimize the frequency of nephrolithiasis events among PwCF.

Compared to prior studies, we identified a much higher prevalence of nephrolithiasis, which is likely explained by several factors. First, we elected to include asymptomatic nephrolithiasis rather than only symptomatic nephrolithiasis events. The primary focus of the study was to determine whether EPI control impacted the development of nephrolithiasis, and so any nephrolithiasis event was included. If asymptomatic stones were not included, the prevalence in our study would have been 20.2%, similar to other cohorts [2,8]. Second, our study population was recruited from an adult CF center and is older than most other series by nearly 10 years [1]. Age is a risk factor for nephrolithiasis in the general population, and the risk of calcium oxalate nephrolithiasis increases with duration of EPI so age may have contributed to the increased prevalence in our study [1,9]. Lastly, several subjects had additional risk factors including small bowel resection, hyperparathyroidism, obesity, hypertension, and diabetes. These comorbidities have not been studied specifically in CF, but are established risk factors for nephrolithiasis in the non-CF population and likely conferred additional risk in our cohort [10].

The majority of stones recovered in our cohort were composed of calcium oxalate, which is consistent with previous observations suggesting that enteric hyperoxaluria is the leading cause of nephrolithiasis among PwCF [1,2]. However, we did not identify an association between prior nephrolithiasis and EPI control. There are several possible explanations for this. First, it may be that EPI directly affects urine oxalate levels and distinct risk factors are required for hyperoxaluria to progress to the development of calcium oxalate nephrolithiasis. Prior studies have shown low urine volume and low urine citrate levels among PwCF, which could facilitate stone formation in the presence of enteric hyperoxaluria [11–14]. Very few subjects in our cohort completed 24 hour urine testing so we are unable to assess for these risk factors. Second, we assessed EPI control at a single point in time and were unable to account for episodes of poor compliance with enzymes which could have contributed to past nephrolithiasis events. As much as possible, we adjudicated EPI control status near any nephrolithiasis event to mitigate this limitation. Lastly, the degree of EPI control was largely determined by patients’ reported symptoms of steatorrhea during clinical encounters with the CF care team, with weight and fat soluble vitamin levels remaining relatively stable in this adult cohort. Since patients may under report symptoms, our EPI control assessment was suboptimal [15]. However, this limited review of EPI symptoms represents the current clinical practice for monitoring EPI control until a standardized monitoring strategy for PERT dosing can be developed and implemented. Such a monitoring strategy should include a more holistic EPI patient reported outcome measure coupled with an easily obtainable biological marker to attempt a more objective representation of EPI control.

Several additional factors contribute to the pathophysiology of enteric hyperoxaluria and could lead to oxalate nephrolithiasis independent of EPI status. In the context of EPI, enteric hyperoxaluria involves a balance between fat intake along with a sufficient dose of PERT, calcium intake, and oxalate intake. In this retrospective design, we could not assess dietary intake but did measure pancreatic enzyme dosages and found no significant difference between PwCF with and without prior nephrolithiasis. Prior studies have also shown that PwCF have diminished levels of *Oxalobacter formigenes,* which can contribute to greater gastrointestinal absorption of oxalate [16,17]. Finally, the strongest association with nephrolithiasis in our study was with prior small bowel resection, which contributes to enteric hyperoxaluria by limiting the absorptive area for fat, delivering more malabsorbed fat to the colon, and facilitating pathologic oxalate reabsorption in the colon [6]. Thus, other factors related to fat digestion may contribute to the risk of calcium oxalate nephrolithiasis in CF, so detailed assessment of EPI status will require prospective study.

Despite the limitations inherent to the retrospective study design and challenges with accurately assessing EPI control status, our study has several strengths. We report one of the largest cohorts of adults with CF with over 3,000 patient-years of follow up, including review of clinic encounters and radiographic studies to assess for asymptomatic stones. In this large cohort, we report that nearly 1 in 3 PwCF will experience a kidney stone at some point in their lifetime. Low BMI, prior alcohol use, and small bowel resection surgery are associated with nephrolithiasis. Future prospective study is warranted to better understand the contribution of dietary intake and EPI control on enteral hyperoxaluria in CF in order to develop a strategy to prevent nephrolithiasis in this high-risk population.

## Supporting information

S1 Table Comparison of clinical factors in subjects with vs. without a history of lung transplant. (DOCX)
