# Supplementary material for: Prevalence and risk factors for nephrolithiasis in adults with cystic fibrosis: A retrospective cohort study
Source: PLoS One. 2026 Jan 6;21(1):e0340293. doi: 10.1371/journal.pone.0340293 (PMC12773813; doi:10.1371/journal.pone.0340293)
Supplement: S1 Table — (DOCX) [file pone.0340293.s001.docx]

**Incidence and prevalence of nephrolithiasis in adults with cystic fibrosis**

**Supplementary Table 1.** Comparison of clinical factors in subjects with vs. without a history of lung transplant.

| Variable | Lung Transplant  N = 20 | No Lung Transplant  N = 87 | p-value |
| --- | --- | --- | --- |
| Age at first event, years, Mean (SD) | 35.9 (11.2) | 29.5 (10.8) | 0.024 |
| BMI at time of event, years, Mean (SD) | 21.0 (3.6) | 23.6 (5.9) | 0.125 |
| Symptomatic | 6 (33.3) | 61 (73.5) | 0.001 |
| Type of stone**  CaOx  Other  Not assessed | 5 (25.0)  1 (5.0)  14 (70.0) | 26 (29.9)  6 (6.9)  55 (63.2) | 0.339 |
| Recurrent stone | 9 (45.0) | 11 (55.0) | 0.523 |
| Intervention | 14 (70.0) | 55 (63.2) | 0.568 |
| Type of intervention  PERT adjustment  Medication to  prevent recurrence  Increased fluid goal  and/or diet change  Invasive procedure | 1 (5.0)  14 (70.0)  4 (20.0)  6 (30.0) | 6 (7.0)  38 (43.7)  29 (33.7)  32 (36.8) | 1.000  0.034  0.233  0.568 |
| Referral to nephrology | 9 (45.0) | 14 (16.1) | 0.012 |
| Referral to urology | 10 (50.0) | 42 (48.3) | 0.889 |
| Use of kidney stone medication  Citrate  Diuretic | 11 (55.0)  7 (35.0) | 32 (36.8)  16 (18.4) | 0.134  0.131 |
| 24hr urine ever | 5 (26.3) | 11 (12.9) | 0.164 |

**Includes n=38 with retrieved stones; p-value = 1.000 for comparing CaOx to Other alone
